# Effects of an Adolescent Sexual and Reproductive Health Intervention on Health Service Usage by Young People in Northern Ghana: A Community-Randomised Trial

**DOI:** 10.1371/journal.pone.0125267

**Published:** 2015-04-30

**Authors:** Gifty Apiung Aninanya, Cornelius Y. Debpuur, Timothy Awine, John E. Williams, Abraham Hodgson, Natasha Howard

**Affiliations:** 1 Navrongo Health Research Centre, Navrongo, Ghana; 2 Kwame Nkrumah University of Science and Technology, Kumasi, Ghana; 3 Research and Development Division, Ghana Health Service, Accra, Ghana; 4 Department of Global Health and Development, London School of Hygiene and Tropical Medicine, London, United Kingdom; Leibniz Institute for Prevention Research and Epidemiology (BIPS), GERMANY

## Abstract

**Background:**

While many Ghanaian adolescents encounter sexual and reproductive health problems, their usage of services remains low. A social learning intervention, incorporating environment, motivation, education, and self-efficacy to change behaviour, was implemented in a low-income district of northern Ghana to increase adolescent services usage. This study aimed to assess the impact of this intervention on usage of sexual and reproductive health services by young people.

**Methods:**

Twenty-six communities were randomly allocated to (i) an intervention consisting of school-based curriculum, out-of-school outreach, community mobilisation, and health-worker training in youth-friendly health services, or (ii) comparison consisting of community mobilisation and youth-friendly health services training only. Outcome measures were usage of sexually-transmitted infections (STIs) management, HIV counselling and testing, antenatal care or perinatal services in the past year and reported service satisfaction. Data was collected, at baseline and three years after, from a cohort of 2,664 adolescents aged 15–17 at baseline.

**Results:**

Exposure was associated with over twice the odds of using STI services (AOR 2.47; 95%CI 1.78–3.42), 89% greater odds of using perinatal services (AOR 1.89; 95%CI 1.37–2.60) and 56% greater odds of using antenatal services (AOR 1.56; 95%CI 1.10–2.20) among participants in intervention versus comparison communities, after adjustment for baseline differences.

**Conclusions:**

The addition of targeted school-based and outreach activities increased service usage by young people more than community mobilisation and training providers in youth-friendly services provision alone.

## Background

### Adolescent sexual and reproductive health needs

Encouraging adolescent sexual and reproductive health (ASRH) service usage is a public health challenge globally. Many governments have pursued strategies to address the specific sexual and reproductive health needs of adolescents since the 1994 International Conference on Population and Development (ICPD) placed ASRH on the global policy agenda [1]. However, the large relative proportion of adolescents in low and middle-income countries and related high rates of HIV, unwanted pregnancy, maternal mortality and unsafe abortion indicate needs for greater improvements in service usage [2, 3]. Many adolescents in sub-Saharan African countries underuse sexual and reproductive health (SRH) services [4–8] due to barriers such as service costs and distance, lack of awareness about where to get contraceptives and STI treatment, embarrassment, lack of confidentiality and privacy, and negative provider attitudes [5, 9–11].

Findings in Ghana are similar to those regionally. Post-ICPD, the Government of Ghana has worked to support adolescents through Adolescent Reproductive Health Policy (2000) and National HIV/AIDS and STIs Policy (2001) initiatives, while Ghanaian health services (GHS) promote youth-friendly policies [12]. However, evidence suggests Ghanaian adolescents still avoid SRH services, particularly due to stigma around premarital sex, while over 750,000 adolescents become pregnant annually [12, 13]. Awusabo-Asare and colleagues found that 2 in 3 young women and 4 in 5 young men with STI symptoms did not seek treatment, while approximately half of unmarried sexually-active female adolescents and over one-third of sexually-active male adolescents did not use contraceptives [14]. Recent surveys found 5.2% female and 3.4% male adolescents reported having experienced STIs, HIV and syphilis prevalence among Ghanaian adolescents was 1.9% and 5.5% respectively, and 8% of female adolescents reported using contraceptives, while 13% of female adolescents had already given birth or were pregnant with their first child [15, 16].

Northern Ghana (i.e. Northern, Upper West and Upper East regions) has poorer economic and health outcomes than the national average, as geographical, historic, and socio-cultural factors have excluded the north from much of Ghana’s economic growth [17]. Research in Ghana’s Upper East Region showed 32% of out-of-school adolescents experienced difficulties accessing HIV testing services [18]. Qualitative research found adolescents were particularly deterred from accessing health services by costs and negative provider attitudes [19]. Karim and others advocated increasing adolescent SRH service usage in Upper East Region [20, 21].

### Interventions to increase ASRH service usage

Several studies document associations between ASRH interventions and adolescent service usage worldwide and in sub-Saharan Africa [22–38]. A UNESCO review indicated that more than 30% of sexuality education programmes increased condom and contraceptive use [39]. A review of ASRH interventions in developing countries found most positively affected adolescent knowledge and attitudes, increasing health service attendance and contraceptive usage [31]. However, interventions varied tremendously and many were not explicitly theoretically grounded. Evaluation methods also varied and often included relatively small sample sizes and time-periods, limiting potential conclusions. Only one large-scale ASRH intervention, in rural Tanzania, was evaluated over a three-year period [40–42].

Only two well-documented evaluations of adolescent SRH interventions have been conducted in Ghana, neither in northern Ghana [27, 43]. A three-month peer-education project, initiated by the Association for Reproductive and Family Health, reported significant changes in reproductive health knowledge, use of contraceptives, willingness to buy contraceptives, and self-efficacy in contraceptive usage [27]. Evaluation of a 12-month SRH intervention by the African Youth Alliance found young women exposed to the intervention were more likely to use modern contraceptives and condoms than those not exposed [43]. However, both were small and offered limited applicability for policy-makers. Thus, policy-makers determined that large-scale ASRH interventions were needed in Ghana and should be evaluated [44, 45].

Responding to the need for the implementation and evaluation of ASRH interventions in Ghana, particularly in the north, the Navrongo Health Research Centre (NHRC) was commissioned to evaluate a three-year community-based ASRH intervention in Kassena-Nankana Districts (KND), northern Ghana. KND was selected as government data indicated poor ASRH indicators, limited adolescent usage of services, and no previous evaluations of interventions to increase adolescent service usage [44–46].

### Aim and objectives

The study aimed to assess whether a community-based ASRH intervention, using a social learning theory approach, was associated with increased adolescent usage of selected services in northern Ghana. Objectives were to: (i) determine whether provision of an adolescent school-based curriculum and peer outreach activities increased adolescent usage of services for antenatal care, perinatal care, STI management, and HIV counselling and testing, in comparison with community mobilisation and youth-friendly health services (YFHS) provider training alone; and (ii) identify changes in reported satisfaction with services among adolescents exposed to community-based activities compared with adolescents exposed to community mobilisation and YFHS alone. Implications for policy and practice were considered.

## Methods

### Study site and population

**Fig 1:**
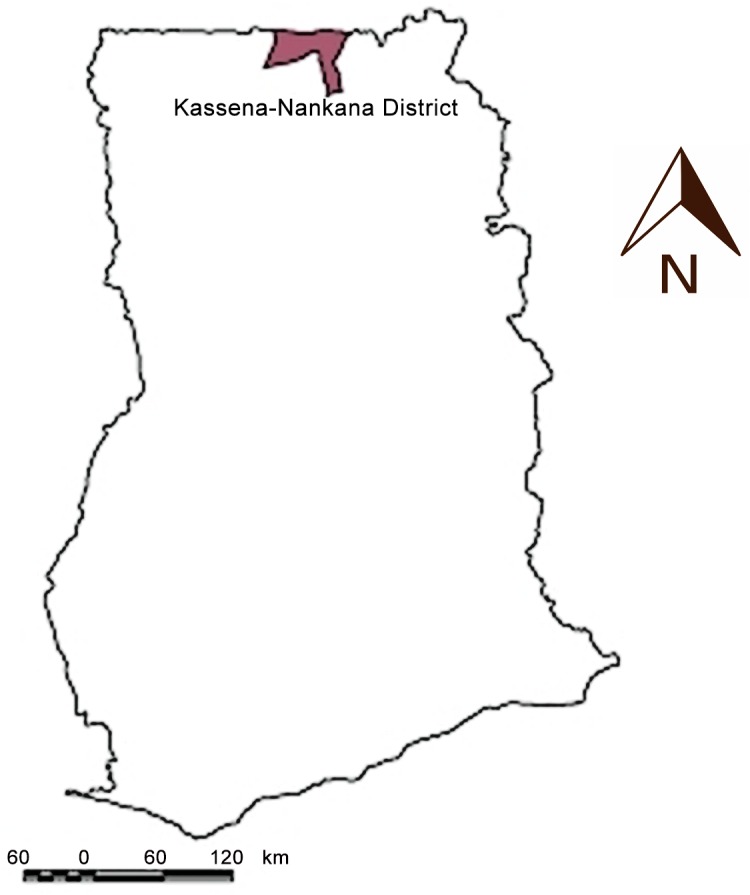
Map of Ghana indicating study area. Source: http://openi.nlm.nih.gov/imgs/rescaled512/2935923_GHA-3-5233-g001.png (14/03/2015)

KND is located in the Upper East Region of Ghana (Fig 1). The 1,675 square kilometre district, split administratively into Kassena-Nankana East and West, has a population of approximately 152,000, 84% rural [47, 48]. The main ethnic groups are Kassena (54%) and Nankana (42%), with the remaining 4% predominantly Builsa [49]. A hospital, five health centres, and 27 community-based health planning and services (CHPS) compounds provide health services. CHPS are a bridge between GHS and communities in healthcare decision-making and community-level services delivery. Adolescents aged 10–19 years are 24% of the district population, 80% of them enrolled in school [48]. A 2003 survey indicated that mean age at sexual initiation was 14 years, 50% of all first sexual encounters among KND adolescents were unprotected, and adolescents under-used SRH services [50]. Health facility attendance records from 2006 indicated that 32% of adolescent visits were STI-related, while 2007 data indicated that adolescent HIV prevalence was 2.7% [15, 44].

### Intervention

Investigators aimed to compare the effectiveness of community-level sexual health education and outreach for improving adolescent SRH service usage additional to YFHS and community mobilisation. The ASRH programme, based on social learning theory principles, aimed to improve adolescent access to and usage of SRH services through four components [51]. All participating communities received: (1) mobilisation, to create a supportive environment, and (2) health-worker training in YFHS approaches, to make services more effective and appealing. Intervention communities additionally received: (3) a school-based sexual health education module, to increase adolescent knowledge and attitudes about healthy sexuality, and (4) out-of-school peer outreach to improve self-efficacy and interaction between young people and health-workers [50]. To increase sustainability, the programme was delivered and supervised through existing systems by government workers trained and supported by five NHRC researchers.

Bandura’s social learning theory, as applied in health and sexuality education, aims to change behaviour through engaging environmental influences, relationships, knowledge-change, and behavioural skills practice [52, 53]:

1. **Community mobilisation (Environment):** A supportive environment was considered crucial for sustainability and encouraged through over fifty meetings and seminars among community stakeholders, including the district assembly, district health personnel, Ghana Education Service officials, the National Youth Council and non-governmental organizations (e.g. Ghana Red Cross, Catholic Relief Services), community chiefs and elders, with a particular focus on working with religious leaders as particularly influential in KND;
2. **Youth-friendly health services (Environment/Relationships):** Activities to make government SRH services more appealing to adolescents included provider consultations and training, promoting dialogue and interaction between adolescents and providers, and assessing health facilities to address barriers to YFHS provision. Nineteen health providers, drawn from both intervention (n = 10) and comparison (n = 9) facilities, attended a Ghana Health Service YFHS training workshop that emphasised changing provider attitudes towards adolescents. Providers were trained and equipped for: (i) syndromic management of STIs and provision of STI drugs; (ii) provision of contraceptives; (iii) friendly and responsive approaches to adolescents, (iv) ASRH counselling, and (v) developing an action plan to promote YFHS at their respective facilities. In intervention communities, a series of outreach events and facility tours were organised for providers to discuss available services with adolescents and promote interaction between providers and adolescents;
3. **School-based SRH education (Knowledge/Behaviour):** The school-based component, a collaboration between the NHRC researchers, the District Health Management Team, and the Ghana Education Service, improved knowledge and skills practice through SRH teaching and learning in Junior High Schools (JHS) with the aim of providing accurate SRH information, building life-skills and changing sexual attitudes and behaviour (e.g. service usage). Methods included in-class participatory teaching and co-curricular SRH activities (e.g. inter-school competitions and debates, video performances, dramas, and role plays, sports). Approximately 75 teachers, in all 25 intervention-area JHS, were trained in a standard SRH curriculum and promotion of co-curricular and extracurricular activities;
4. **Peer outreach (Knowledge/Behaviour):** The peer component trained and supervised young people to provide SRH information and peer counselling to out-of-school adolescents. Approximately 156 peer-workers were recruited (i.e. 12 per intervention community). Each peer-worker had a defined catchment area in which to reach young people with relevant SRH information and provide health facility referrals. Methods included one-to-one counselling sessions, group talks, and recreational activities (e.g. dramas, films, sporting events), and 41 adults were trained to support and legitimise peer-worker activities in communities.

### Study design and sampling

The design chosen was a community-randomised trial. For study purposes, KND was divided into 26 communities with an average population of 4,500 young people each (range 861–12,392). Communities were the unit of allocation, serving as clusters randomly allocated as intervention or comparison, while analysis subjects were young people living in study communities [54]. After a ten-month intervention pilot in two communities, remaining communities were assigned using simple random allocation with sealed envelopes. First, communities were identified by zone and size. Second, names were sealed in opaque envelopes. Third, representatives from Ghana Education Service and the District Health Management Team drew envelopes for intervention or comparison alternately. Thus, 11 additional communities were randomly allocated to receive the intervention (i.e. all programme components), covering a population of 36,840 young people [50], while 13 served as comparison sites (i.e. receiving YFHS and mobilisation components only). All young people aged 10–24 years living in intervention communities were potential beneficiaries, while young people in comparison communities benefitted from community mobilisation and health-worker YFHS training.

Study outcomes were reported health service usage within the past 12 months for STI management (i.e. diagnosis, treatment), HIV counselling and testing, antenatal care, perinatal services (e.g. delivery, postnatal care), and reported satisfaction with services received—as these were considered most crucial by service providers. The exposure variable was residency in an intervention community, as all adolescents were expected to be exposed. All residents aged 15–17 in 2005 were potentially eligible for survey cohort inclusion. Participants were selected through random sampling of district compounds from the Navrongo Health and Demographic Surveillance System (NHDSS) database in 2005 and retargeted for interview in 2008. Sample size was calculated assuming an intracluster correlation coefficient of 0.10, to account for a 33% difference in between-group outcomes with a response rate of 60% at follow-up, a 1.4% design effect, 95% significance, and 90% power [45].

### Data collection and analysis

Data was collected at baseline (2005) and endline (2008) via cohort survey. Trained interviewers, the same gender as participants, visited each sampled compound, identified eligible adolescents, obtained parental and participant consent, and interviewed in one of the three local languages (i.e. Kasem, Nankam, Buili) at convenient locations for participants. Questionnaires, developed for male and female participants based on study objectives and similar research, were piloted in two non-study communities. Data was collected on socio-demographic characteristics, and SRH knowledge, attitudes, and reported practices during the past year (e.g. sexual norms, gender-based violence, contraceptive usage, health service usage).

Data was entered, cleaned and checked for inconsistencies using Microsoft FoxPro 6.0 and analysed using Stata12.0. Primary analysis was by intention-to-treat, defined by a participant’s residence during initial community allocation in 2005, with clustering accounted for using random effects. Odds ratios for changes between study arms from baseline to endline were calculated using random effects Poisson regression techniques. *A priori* confounders included household income, age, gender, and baseline service usage. Reporting was guided by the CONSORT extension recommended by Campbell *et al* [54, 55].

### Ethics

Approval was provided by the Navrongo Health Research Centre Institutional Review Board and ethics committees of the Ghana Health Service and the London School of Hygiene & Tropical Medicine in the United Kingdom. Written informed consent was obtained from guardians and participants. Anonymity and confidentiality were safeguarded by using identification numbers for names and locations, storing completed questionnaires in locked files, and conducting interviews in locations requested by participants.

## Results

### Demographics

**Fig 2:**
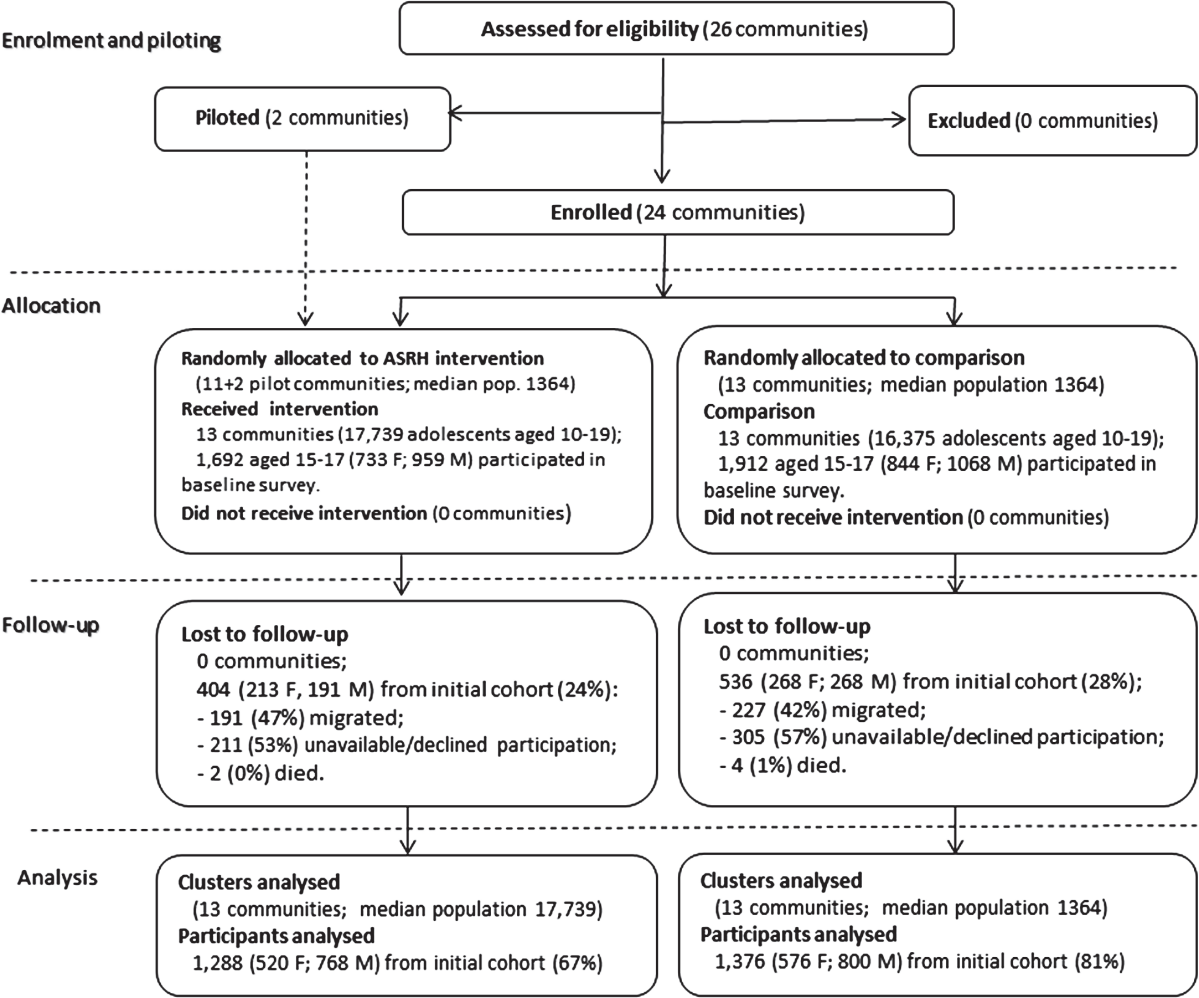
Trial profile.

Fig 2 shows the trial profile. A total of 2,664 adolescents participated in the 2008 survey, 1,288 in intervention (i.e. 520 female, 768 male) and 1,376 in comparison (i.e. 576 female, 800 male) sites. A 26% loss-to-follow-up was similar between intervention (24%) and comparison (28%) sites.

Table 1 shows frequencies of demographic characteristics at baseline, comparing adolescents in intervention and comparison sites, disaggregated by gender. Average participant age was 16 at baseline in both intervention and comparison communities. Most participants attended primary (52%) or junior high school (35%). Significant demographic differences between intervention and comparison participants included a higher percentage of comparison participants attending primary school (39% versus 36%) and identifying as Catholic (49% versus 44%), and a lower percentage identifying as Muslim (6% versus 11%). No significant differences were found between communities in sexual experience among either boys or girls or pregnancy experience and parity among girls.

**Table 1:** Demographic characteristics at baseline, comparing intervention to comparison adolescents disaggregated by gender.

Variables, n (%)	Intervention			Comparison		
**2005 Baseline survey**	**Female** (n = 733)	**Male** (n = 959)	**All** (n = 1692)	**Female** (n = 844)	**Male** (n = 1068)	**All** (n = 1912)
**Mean age**	16.0	16.0	16.0	16.0	16.0	16.0
**Education**						
Attending Primary	47 (6.4)	558 (58.2)	605 (35.8)	54 (6.4)	683 (64.0)	737 (38.5)
Attending Junior High School	316 (43.1)	264 (27.5)	580 (34.3)	435 (51.5)	259 (24.3)	694 (36.3)
Attending Senior High School	331 (45.2)	44 (4.6)	375 (22.2)	334 (39.6)	11 (1.0)	345 (18.0)
Not attending school	39 (5.3)	93 (9.7)	132 (7.8)	21 (2.5)	115 (10.8)	136 (7.1)
**Religion**						
Catholic	386 (52.7)	364(38.0)	750 (44.3)	496 (58.8)	447 (41.9)	943 (49.3)
Other Christian	217 (29.6)	165(17.2)	382 (22.6)	281 (33.3)	217 (20.3)	498 (26.0)
Muslim	96 (13.1)	102(10.6)	198 (11.7)	18 (2.1)	103 (9.6)	121 (6.3)
Traditional	24 (3.3)	99(10.3)	123 (7.3)	32 (3.8)	49 (4.6)	81 (4.24)
No religion	10 (1.4)	229(23.9)	239 (6.3)	17 (2.0)	252 (23.6)	269 (14.1)
**Living with mother**	535 (72.9)	764(79.7)	1299 (76.8)	687 (81.4)	910 (85.2)	1597 (83.5)
**Ever had sexual intercourse**	123 (16.8)	125 (13.0)	248 (14.7)	95 (11.3)	106 (9.9)	201 (10.5)
**Ever pregnant/impregnated**	32 (4.4)	2 (0.2)	34 (2.0)	28 (3.3)	1 (0.1)	29 (1.5)
**Ever given birth**	18 (2.5)	..	9 (1.1)	20 (2.4)	..	20 (1.0)

### Service usage

**Fig 3:**
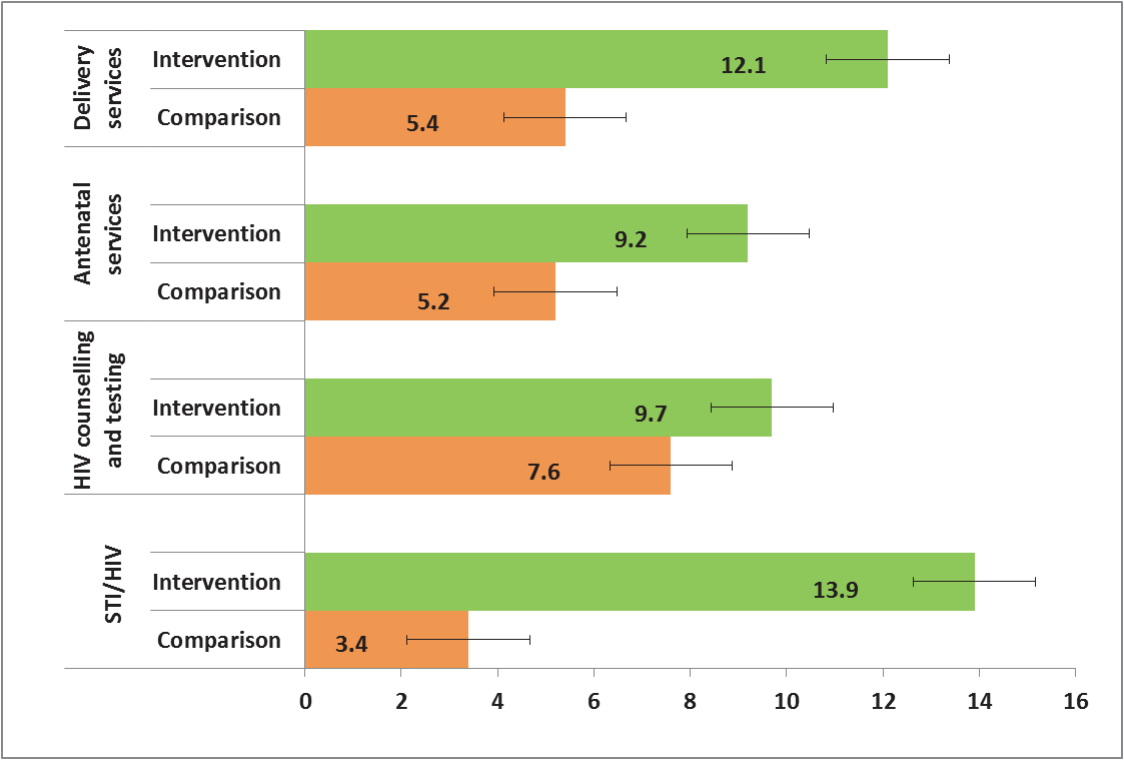
Percentage change in service usage. Percentage differences between baseline and endline in intervention and comparison sites.

Table 2 shows reported services usage during the past twelve months at baseline and endline. Usage of three of the four services increased among intervention adolescents, most notably STI services (Fig 3). Reported usage of STI services increased from 3% to 17% among intervention adolescents versus 5% to 8% among comparison adolescents, so young people in intervention areas had more than twice the odds of using STI services than those in comparison areas at endline (OR 2.47, adjusted for baseline usage; 95%CI 1.78–3.42). More young men than young women used STI services at endline (i.e. 64% versus 36% in intervention communities and 57% versus 43% in comparison communities respectively).

**Table 2:** Frequency of service usage, change over time and odds ratios with 95% confidence intervals, comparing intervention to comparison responses at baseline and endline.

Service usage	Arm	Baseline (2005)				Endline (2008)				Change (%)	OR (95%CI)
		N	n (%)	Female	Male	N	n (%)	Female	Male		
**STI management**	Comparison	1912	86 (4.5)	51 (59.3)	35 (40.7)	1376	109 (7.9)	47 (43.1)	62 (56.9)	3.4	
	Intervention	1692	43 (2.5)	32 (74.4)	11 (25.6)	1288	214 (16.6)	77 (36.0)	137 (64.0)	13.9	2.47 (1.78, 3.42)**
**HIV counselling and testing**	Comparison	1912	73 (3.8)	46 (63.0)	27 (37.0)	1376	157 (11.4)	56 (35.7)	101 (64.3)	7.6	
	Intervention	1692	55 (3.3)	32 (58.2)	23 (41.8)	1288	167 (13.0)	60 (35.9)	107 (64.1)	9.7	1.16 (0.85,1.58)
**Antenatal**	Comparison	1912	57 (3.0)	36 (63.2)	21 (36.8)	1376	113 (8.2)	75 (66.4)	38 (33.6)	5.2	
	Intervention	1692	53 (3.1)	41 (77.4)	12 (22.6)	1288	159 (12.3)	101 (63.5)	58 (36.5)	9.2	1.56 (1.10, 2.20)*
**Peri/Postnatal**	Comparison	1912	63 (3.3)	41 (65.1)	22 (34.9)	1376	120 (8.7)	74 (61.7)	46 (38.3)	5.4	
	Intervention	1692	52 (3.1)	46 (88.5)	6 (11.5)	1288	196 (15.2)	94 (52.0)	102 (48.0)	12.1	1.89 (1.37, 2.60)**

**NB**:

*p<0.05;

**p<0.001.

Reported usage of HIV counselling and testing increased from 3% to 13% among intervention versus 4% to 11% among comparison adolescents. Thus, usage was lower among intervention adolescents at baseline and slightly higher at endline (OR 1.16, adjusted for baseline usage; 95%CI 0.85–1.58) though this difference was not significant. Young men were more likely to use CT services than young women in both intervention and comparison areas at endline (64% versus 36% respectively).

Reported usage of antenatal services increased from 3% to 12% among intervention versus 3% to 8% among comparison adolescents. Thus, antenatal usage also increased from a lower baseline frequency among intervention adolescents to 56% higher odds of using antenatal services than comparison adolescents at endline (OR 1.56, adjusted for baseline ANC usage; 95%CI 1.10–2.20). More young women than young men reported using antenatal services at endline, though gender differences were smaller among intervention adolescents (i.e. 64% versus 58% and 66% versus 34% in intervention and comparison areas respectively).

Reported usage of peri/postnatal services increased from 3% to 15% among intervention versus 3% to 9% among comparison adolescents. Thus, intervention adolescents who had 89% higher odds of attending delivery and postnatal services than comparison adolescents at endline (OR 1.89, adjusted for baseline usage; 95%CI 1.37–2.60). As with antenatal services, more young women than young men reported using peri/postnatal services at endline, though gender differences were smaller among intervention adolescents (i.e. 52% versus 48% and 62% versus 38% in intervention and comparison areas respectively).

No obvious differences in SRH service usage between intervention and comparison adolescents were found by educational participation (Table 3). As numbers not attending school were very low (i.e. less than 7% of the study population), most service-users were secondary-school students. For example, in intervention areas at endline, 61% of antenatal attendees were secondary-school students compared with 32% primary students and 8% out-of-school adolescents (Table 3).

**Table 3:** SRH service usage among school-attending and out-of-school adolescents at baseline and endline.

Service usage	Arm	Baseline (2005)				Endline (2008)			
		n (%)	Primary (%)	Secondary (%)	No school (%)	n (%)	Primary (%)	Secondary (%)	No school (%)
**STI management**	Comparison	86 (4.5)	32 (37.2)	53 (61.6)	1 (1.2)	109 (7.9)	16 (14.7)	89 (81.7)	4 (3.7)
	Intervention	43 (2.5)	16 (37.2)	24 (55.8)	3 (7.0)	214 (16.6)	33 (15.4)	172 (80.4)	9 (4.2)
**HIV counselling and testing**	Comparison	73 (3.8)	41 (56.2)	30 (41.1)	2 (2.7)	157 (11.4)	38 (24.2)	114 (72.6)	5 (3.2)
	Intervention	55 (3.3)	31 (56.4)	18 (32.7)	6 (10.9)	167 (13.0)	35 (21.0)	124 (74.3)	8 (4.8)
**Antenatal**	Comparison	57 (3.0)	23 (40.4)	26 (45.6)	8 (14.0)	113 (8.2)	40 (35.4)	62 (54.9)	11 (9.7)
	Intervention	53 (3.1)	24 (45.3)	21 (39.6)	8 (15.1)	159 (12.3)	50 (31.5)	97 (61.0)	12 (7.5)
**Peri/Postnatal**	Comparison	63 (3.3)	22 (34.9)	35 (55.6)	6 (9.5)	120 (8.7)	28 (23.3)	83 (69.2)	9 (7.5)
	Intervention	52 (3.1)	26 (50.0)	19 (36.5)	7 (13.5)	196 (15.2)	39 (19.9)	145 (74.0)	12 (6.1)

NB: Due to very low numbers of out-of-school adolescents, further analysis was not conducted.

### Satisfaction and suggested service improvements

Adolescents who visited health facilities were asked whether they were satisfied with services received (Table 4A). Reported satisfaction increased from 18% to 43% among intervention adolescents versus 17% to 28% among comparison adolescents. Thus, intervention adolescents had 92% higher odds of reporting satisfaction with services than comparison adolescents at endline (OR 1.92, adjusted for baseline satisfaction; 95%CI 1.63–2.26). No gender differences were found in reported service satisfaction.

**Table 4:** Frequency of reported satisfaction with services and suggested improvements, comparing intervention to comparison responses at baseline and endline.

4a. Service satisfaction	Arm	Baseline (2005)				Endline (2008)				Change (%)
		N	n (%)	Female	Male	N	n (%)	Female	Male	
Reported satisfaction*	Comparison	1912	330 (17.3)	146 (44.2)	184 (55.8)	1376	390 (28.3)	180 (46.2)	210 (53.8)	11.0
	Intervention	1692	304 (18.0)	141 (46.4)	163 (53.6)	1288	556 (43.2)	250 (45.0)	306 (55.0)	25.2
**4b. Suggested improvements** ^**a**^											
Friendly staff	Comparison	1912	1 (0.1)	1 (100)	0 (0)	1376	9 (0.7)	5 (55.6)	4 (44.4)	0.6
Intervention	1692	1 (0.1)	1 (100)	0 (0)	1288	10 (0.8)	8 (80.0)	2 (20.0)	0.7
Privacy	Comparison	1912	2 (0.1)	2 (100)	0 (0)	1376	4 (0.3)	2 (50.0)	2 (50.0)	0.2
Intervention	1692	1 (0.1)	0 (0)	1 (100)	1288	9 (0.7)	7 (77.8)	2 (22.2)	0.6
Convenient hours	Comparison	1912	1 (0.1)	0 (0)	1 (100)	1376	6 (0.4)	3 (50.0)	3 (50.0)	0.3
Intervention	1692	1 (0.1)	0 (0)	1 (100)	1288	3 (0.2)	1 (33.3)	2 (66.7)	0.1
Same sex providers	Comparison	1912	1 (0.1)	0 (0)	1 (100)	1376	2 (0.2)	1 (50.0)	1 (50.0)	0.1
Intervention	1692	0 (0)	0 (0)	0 (0)	1288	0 (0)	0 (0)	0 (0)	0.0
Drug availability	Comparison	1912	4 (0.2)	0 (0)	4 (100)	1376	23 (1.7)	15 (65.2)	8 (34.8)	1.5
Intervention	1692	5 (0.3)	4 (80)	1 (20.0)	1288	35 (2.7)	21 (60.0)	14 (14.0)	2.4
Short waiting times	Comparison	1912	2 (0.1)	0 (0)	2 (100)	1376	5 (0.4)	5 (100)	0 (0)	0.3
Intervention	1692	0 (0)	0 (0)	0 (0)	1288	9 (0.7)	5 (55.6)	4 (44.4)	0.7
Confidentiality	Comparison	1912	0 (0)	0 (0)	0 (0)	1376	2 (0.2)	1 (50.0)	1 (50.0)	0.2
Intervention	1692	1 (0.1)	0 (0)	1 (100)	1288	8 (0.6)	7 (87.5)	1 (12.5)	0.5

**NB**:

*OR 1.92 (95%CI 1.63–2.26, significant at p<0.05 level).

^a^ Multiple answers possible. Those suggesting improvements numbered 20 at baseline (i.e. 11/1,692 intervention versus 9/1,912 comparison) and 99 at endline (i.e. 54/1,288 intervention versus 45/1,376 comparison).

Participants reporting dissatisfaction were asked for improvement suggestions, which 20 provided at baseline and 99 at endline (Table 4B). Dissatisfied participants wanted improvement in drug availability, staff friendliness, privacy and convenient hours at endline.

## Discussion

The intervention resulted in increases in reported adolescent usage of STI, antenatal and perinatal services, additional to those resulting from strengthening awareness and YFHS provision in all communities. Baseline usage was sufficiently low that any increase is encouraging, though the cost-effectiveness of intervention components should be included in policy-related decisions [36, 37, 56–58]. Early detection and treatment of STIs and HIV is vital to adolescent health, while antenatal and perinatal services increase healthy outcomes for adolescent mothers and babies [59, 60]. Incorporation of social learning theory into intervention design, through influencing community environments, relationship-building, knowledge gains, and behaviour modelling and practice, appeared to improve on behaviour-change approaches focused on increasing knowledge and awareness [61]. This study demonstrated that a school-based and peer-outreach interventions were feasible in this setting and could therefore be used for future interventions in KND [62–64].

The intervention had no significant effect on adolescent usage of HIV counselling and testing services, the relatively low usage of which may reflect generally low community usage or fears of confirming HIV status [12]. Stigma and discrimination could have affected adolescent services usage, as found in literature on SRH services uptake [2, 5, 9]. As Doyle *et al* noted in Tanzania, youth interventions integrated within intensive, community-wide risk reduction programmes may be more successful than ASRH interventions and should be evaluated [65]. Young men were more likely to use STI and HIV testing services than young women, suggesting perhaps they felt greater need for these services, and future health messages could emphasis the benefits to young women of knowing their status [66]. Young women in intervention communities used antenatal services significantly more frequently than young men, indicating additional efforts may be warranted to encourage male involvement [67]. Lower service usage among primary-school versus secondary-school participants was largely explainable by age. Lower usage among out-of-school participants appeared reasonable given the low proportion of out-of-school adolescents in the KND study sample.

Adolescents in intervention areas reported more satisfaction with SRH services, as similarly noted by Teijlingen and colleagues in Nepal [56]. Generally, young men reported more satisfaction than did young women in both intervention and comparison areas. Though total numbers of dissatisfied adolescents were small, it is important that the major service recommendation in both groups was increased drug availability. This supports the literature that programmes to increase service usage depend on the quality of services available. Findings also support research indicating adolescents may avoid SRH services where confidentiality and privacy are not guaranteed, provider attitudes are perceived as negative, or waiting times too long [68].

### Limitations

As the intervention consisted of four components, it was not possible in this study to determine which component or components most influenced results. Other potential limitations include loss to follow-up, recall bias and unmeasured confounding. Loss to follow-up was primarily due to migration and not being present when interviewers visited, with no major differences between intervention and comparison communities, so was unlikely to have changed study results significantly. Recall bias may have underestimated some service usage, as participants were asked to recall usage over a twelve-month period. While some unmeasured confounding is possible in any large study, this was expected to be similar in both groups [69–71]. Data collection was quantitative, so reasons for responses could not be explored. Despite limitations, demonstration of increased service usage over a three-year period by adolescents within a rural, resource-constrained, developing country context supports and contributes to the ASRH service-usage literature [25, 29, 72].

## Conclusion

Social learning approaches aim to work through changing knowledge, skills, relationships, and environmental influences [51, 52], helping explain why inclusion of school and community outreach and peer-based activities was more effective than YFHS alone. To enhance adolescent usage of SRH services, health service managers in Ghana should continue developing YFHS, with additional focus on sufficient drug supply, privacy and confidentiality, staff friendliness, and convenient hours [73]. Recognising that ASRH and wellbeing require more than a one-dimensional health services strengthening approach, policy-makers in Ghana could further develop school and peer-based approaches that increase environmental support and adolescent self-efficacy [74–76]. Such an approach was positively associated with increased service usage in this study and is supported by the ASRH literature [65, 77, 78].
